# *Cladosporium cladosporioides* and *Cladosporium pseudocladosporioides* as potential new fungal antagonists of *Puccinia horiana* Henn., the causal agent of chrysanthemum white rust

**DOI:** 10.1371/journal.pone.0170782

**Published:** 2017-01-31

**Authors:** David Eduardo Torres, Reyna Isabel Rojas-Martínez, Emma Zavaleta-Mejía, Patricia Guevara-Fefer, G. Judith Márquez-Guzmán, Carolina Pérez-Martínez

**Affiliations:** 1 Instituto de Fitosanidad, Colegio de Postgraduados, Montecillo, Texcoco, México; 2 Departamento de Ecología y Recursos Naturales, Facultad de Ciencias, Universidad Nacional Autónoma de México, Ciudad de México, Distrito Federal, México; 3 Departamento de Biología Comparada, Facultad de Ciencias, Universidad Nacional Autónoma de México, Ciudad de México, Distrito Federal, México; Universita degli Studi di Pisa, ITALY

## Abstract

*Puccinia horiana* Hennings, the causal agent of chrysanthemum white rust, is a worldwide quarantine organism and one of the most important fungal pathogens of *Chrysanthemum* × *morifolium* cultivars, which are used for cut flowers and as potted plants in commercial production regions of the world. It was previously reported to be controlled by *Lecanicillium lecanii*, *Cladosporium sphaerospermum*, *C*. *uredinicola* and *Aphanocladium album*, due to their antagonistic and hyperparasitic effects. We report novel antagonist species on *Puccinia horiana*. Fungi isolated from rust pustules in a commercial greenhouse from Villa Guerrero, México, were identified as *Cladosporium cladosporioides* and *Cladosporium pseudocladosporioides* based upon molecular analysis and morphological characters. The antagonism of *C*. *cladosporioides* and *C*. *pseudocladosporioides* on chrysanthemum white rust was studied using light and electron microscopy *in vitro* at the host/parasite interface. *Cladosporium cladosporioides* and *C*. *pseudocladosporioides* grew towards the white rust teliospores and colonized the sporogenous cells, but no direct penetration of teliospores was observed; however, the structure and cytoplasm of teliospores were altered. The two *Cladosporium* spp. were able to grow on media containing laminarin, but not when chitin was used as the sole carbon source; these results suggest that they are able to produce glucanases. Results from the study indicate that both *Cladosporium* species had potential as biological control agents of chrysanthemum white rust.

## Introduction

Chrysanthemum white rust (CWR), caused by *Puccinia horiana* Hennings is one of the most destructive diseases of *Chrysanthemum* × *morifolium* worldwide [1,2,3]. Due to its economic importance to floriculture, it is classed as a quarantine disease by the European and Mediterranean Plant Protection Organization (EPPO), Inter-African Phytosanitary Council (IAPSC), Andean Community (CAN) and North American Plant Protection Organization (NAPPO), and is also listed as a regulated pest by the International Plant Protection Convention (IPPC). Currently it is present in most chrysanthemum producing areas throughout the world and can cause important losses [3,4,5].

*Puccinia horiana* is an obligate biotrophic fungus and microcyclic rust, affecting ten different *Chrysanthemum* species [6,7,8,9]. It produces teliospores, which germinate without a period of dormancy and release basidiospores as the infective propagules. The basidiospores are easily spread by wind and can infect neighboring plants in conditions of high humidity and cool temperatures [10, 11]. On the lower surface of the leaves, these teliospores form yellowish to pinkish immature pustules that become the characteristic white color once they mature [6,4,10].

When disease symptoms appear, they often do so suddenly and on a large scale in greenhouses, leaving little or no time for the farmers to take control actions. This leads to an ecologically and economically suboptimal timetable for the preventive application of fungicides [1,4,12]. Reports of fungicide-resistant strains show that preventive application with some fungicides is not effective [13,14], and this has prompted a search for alternative management solutions such as biological control [4,15,16,17].

*Cladosporium* Link is one of the most common genera of fungi occurring on various substrates and includes species with diverse lifestyles. Nowadays, after several taxonomic revisions, most saprobe lifestyles are included in the genus [18]. Besides saprophytic behavior, antagonism to pathogenic fungal species has been described. Some of the most common examples come from the relationship between *Cladosporium* spp. and rust pathogens [19,20], such as: *C*. *uredinicola* parasitizing *Puccinia violae*, *P*. *puta* and *Cronartium fusiforme* [21,22,23]; *C*. *aecidicola* on *Melampsora medusae* [24]; *C*. *tenuissimum* parasitizing *Uromyces appendiculatus*, *Cronartium flaccidum* and *Peridermium pini* [25,26,27]; *C*. *gallicola* on *Endocronartium harknessii* [28]; and *C*. *cladosporioides* parasitizing *Venturia inequalis* and *Puccinia striiformis* f.sp. *tritici* [29,30]. On *Puccinia horiana* only *C*. *uredinicola*, *C*. *sphaerospermum* and *Cladosporium* sp. have been previously reported [15, 17].

In the present study, we found novel species potentially antagonistic towards *P*. *horiana*. Based on morphological characteristics and molecular data, two species not previously reported on *P*. *horiana* were identified. Additionally, we investigated in detail the *in vitro* interaction between the two antagonists and CWR through light and scanning electron microscopy. Our results indicate that the isolates identified had potential as biological control agents of chrysanthemum white rust.

## Materials and methods

### Isolation, purification and morphological determination

Leaves from 30 different 30-day-old chrysanthemum plants with pustules of *P*. *horiana*, extensively colonized by a gray fungus, were collected from a commercial greenhouse with permission and collaboration of the owner in Villa Guerrero, Estado de México, México, in January 2014. Specific permission was not required, based on the epidemiological status of the chrysanthemum white rust in México. Also, this study did not involve endangered or protected species.

Gray dusty mycelia were removed from *P*. *horiana* teliospores with a sterilized needle and transferred to synthetic PDA medium (Bioxon, Mexico). After incubation at 24°C for 5 days, a spore suspension was prepared with sterilized water and transferred to water-agar medium. After incubation for 48h at 24°C, single-spore mycelia were picked off and transferred to PDA medium to obtain pure cultures.

Morphological characteristics were determined following the standardized methodology of Schubert *et al*. [31] for identification of the *Cladosporium* species. Colonies grown, for 5 days at 24°C in the dark, on synthetic-nutrient-limited media (SNA) plates, were used for morphological and morphometric observations of conidia, ramoconidia and conidiophores, using a photomicroscope Provis AX70 (Olympus, USA). Colony characteristics were determined after growing on PDA (Synthetic Potato-Dextrose-Agar; Bioxon, Mexico), MEA (Malt-Extract Agar; Bioxon, Mexico) and OA (Oat Agar), for 14 days at 24°C in the dark.

### Molecular determination

#### DNA extraction and PCR amplification

DNA was extracted from mycelium and spores taken from PDA cultures using the method described by Falcon and Valera [32]. DNA concentration was determined using a NanoDrop N100 spectrophotometer. For PCR amplification, the stock solution was diluted to 90 ng/mL. Partial gene sequences were amplified as described by Bensh *et al*. [18] for internal transcribed spacers (ITS), actin (ACT) and translation elongation factor (EF-1α), using ITS1 (5´-TCCGTAGGTGAACCTGCGG-3´) and ITS4 (5´-GCTGCGTTCTTCATCGATGC-3´) from White *et al*. [33], ACT-512F (5´-ATGTGCAAGGCCGGTTTCGC-3´) and ACT-83R (5´-TACGAGTCCTTCTGGCCAT-3´), EF1-728F (5´-CATCGAGAAGTTCGAGAAGG-3´) and EF1-986R (5´-TACTTGAAGGAACCCTTACC-3´) from Carbone and Cohn [34]. The primers were synthesized by Instituto de Biotecnología, UNAM (Cuernavaca, México). Thermal cycle conditions and PCR mixtures for PCR amplification were those reported by Bensh *et al*. [18], using thermal cycler TC3000 (Techne, USA) and Taq Polimerase (Biotechmol, Mexico). Five mL of the PCR product was electrophoresed on a 1.5% agarose gel in 1% TBE buffer (0.089 M Tris-borate, 0.089 M boric acid and 0.002 M EDTA) for 45 min at 90 V and stained with ethidium bromide. Bands were detected under UV light in a Gel Documentation and Image Analysis System Geldoc 2000 (BioRad, USA). The PCR products were purified by Wizard SV gel and a PCR clean-up system Kit (Promega, USA), and sequenced at Instituto de Biotecnología, UNAM (Cuernavaca, México).

#### Sequence analysis

The three gene sequences from each antagonist, *Cladosporium cladosporioides* and *C*. *pseudocladosporioides*, were aligned with the sequences available in GenBank (NCBI, USA). Our sequences were manually edited by CLCbio (Qiagen, USA). Sequence data obtained from Bensh *et al*. [18,35] were used as reference data for the alignments (Table 1). Multiple alignments were performed by ClustalW software and best nucleotide model determinate by jModelTest v. 2.1.7 [36] using BIC criteria for each locus and then incorporating it in the analysis. A Bayesian phylogenetic inference tree was generated based on data from each partition sequence of the three genes on BEAST v.1.8.1 [37] and Markov Chain Monte Carlo analysis, from four chains started from random tree topology and taken over 80 000 000 generations. Three independent runs were combined by LogCombiner v.1.8.1. Trees were saved each 1 000 generations, resulting in 80 001 saved trees. Using Tracer v.1.6, burn-in was set at 15 000 000 generations, after which the likelihood values were stationary. The coalescent algorithm with GTR+G+I substitution model and a lognormal uncorrelated relaxed clock was selected for the data. Maximum clade credibility tree was visualized by Fig Tree v. 1.4.2. For the stability and robustness of each species, Neighboring-Joining analysis was performed for each data partition, using MEGA 6.0 [38] and 1000 replications using bootstrap. The ITS region has limited resolution for species in *Cladosporium*, therefore results for the ACT and EF-1α regions were used for comparison of clade stability (S1 Fig).

**Table 1:** *Cladosporium* isolates included in the sequence analysis.

Species	Accession number	GenBank numbers (ITS, EF-1α, ACT)			Substrate	Country	Reference
*C*. *cladosporioides*	DETSC1A	KT877404	KT887880	KT721703	*Puccinia horiana*	Mexico	This work
	DETSC1B	KT877405	KT887881	KT721704	*Puccinia horiana*	Mexico	This work
	CBS113738	HM148004	HM148245	HM148491	*Grape blossom*	USA	[35]
	CBS143.35	HM148011	HM148252	HM148498	*Pisum sativum*	South Africa	[35]
	CBS674.82; CBS 320.87	HM148014	HM148255	HM148501	*Gossypium seeds*	Israel	[35]
	CBS11398	HM148024	HM148265	HM148511	*Phragmidium griseum on Rubus crataegifolius*	South Korea	[35]
	CPC12762	HM148030	HM148271	HM148517	*Spinacia oleracea seeds*	USA	[35]
	CPC12852	HM148033	HM148274	HM148520	*Eucalyptus sp*.	Australia	[35]
	CPC14705	HM148050	HM148291	HM148537	*Phyllactinia sp*. *Chaesmothecia on Fraxinus rhynchophylla*	South Korea	[35]
	CPC13220	HM148054	HM148296	HM148541	*Lychen on Acer platanoides*	Germany	[35]
	CPC14238	HM148055	HM148297	HM148542	*Sambucus nigra fruits*	Netherlands	[35]
	CBS306.84	HM148057	HM148299	HM148544	*Puccinia allii*	UK	[35]
	CPC13867	HM148059	HM148301	HM148546	*Leptosphaeria sp*.	South Africa	[35]
	CBS113746	HM148061	HM148303	HM148548	*Cherry fruits*	USA	[35]
	CPC13362	HM148063	HM148305	HM148550	*Paeonia obovata*	Germany	[35]
	CBS112388^1^	HM148003	HM148244	HM148490	*Indoor air*	Germany	[18,35]
*C*. *pseudocladosporioides*	DETSC03	KT877407	KT887883	KT887879	*Puccinia horiana*	Mexico	This work
	CBS117134	HM148156	HM148400	HM148645	*Cloud*	-	[35]
	CBS117153	HM148157	HM148401	HM148646	*Paeonia sp*. *leaves*	Germany	[35]
	CBS 125993^2^	HM148158	HM148402	HM148647	*Outdoor air*	Netherlands	[18,35]
	CBS 176.82	HM148162	HM148406	HM148651	*Pteridium aquilinum*	Romania	[35]
	CBS 574.78A	HM148163	HM148407	HM148652	*Melampsoporidium betulae*	Russia	[35]
	CBS 574.78B	HM148164	HM148408	HM148653	*Melampsoporidium betulae*	Russia	[35]
	CPC 11392	HM148166	HM148410	HM148655	*Chrysanthemum coronarium var*. *spatiosum*	South Korea	[35]
	CPC 11841	HM148168	HM148412	HM148657	*Phalaris aquatica leaves*	New Zeland	[35]
	CPC 12850	HM148169	HM148413	HM148658	*Rotten wood*	USA	[35]
	CPC 13488	HM148171	HM148415	HM148660	*Vernonia sp*.	Brazil	[35]
	CPC 14295	HM148188	HM148432	HM148677	*Soil*	Chile	[35]
	CPC 14357	HM148189	HM148433	HM148678	*Food*, *Coffee leaves*	Uganda	[35]
	CPC 14992	HM148192	HM148436	HM148681	*Eucalyptus sp*.	Indonesia	[35]
	CPC 13992	HM148174	HM148418	HM148663	*Coffee tree*	USA	[35]
*C*. *delicatulum*	DETSC02	KT877406	KT887882	KT887878	*Puccinia horiana*	Mexico	This work
	CBS 126344; CPC 11389^3^	HM148081	HM148325	HM148570	*Tilia cordata*	Germany	[18,35]
*C*. *sphaerospermum*	CBS193.54	DQ780343	EU570261	EU570269	-	-	[39]
*C*. *herbarum*	CBS 121621; CPC 12177	EF679363	EF679440	EF679516	-	-	[31]
*C*. *tenuissimum*	CBS125995; CPC14253	HM148197	HM148442	HM148687	-	-	[18]
*Cercospora beticola*	CBS116456	AY840527	AY840494	AY840458	-	-	[18,35]

ACT: partial actin gene, EFα1: partial translation elongation 1-α gene, ITS: internal transcribed spacer with 5.8 rRNA gene.

^**1**^ Ex-type from neotype;

^**2**^ Ex-type from holotype;

^**3**^ Reference strain

### Antagonism assay

For the antagonism test, leaves infected (severity up 30%) and non-infected by chrysanthemum white rust, were collected from 20 different 30-day-old *Chrysanthemum* × *morifolium* cv. Polaris plants in a commercial greenhouse at Texcoco, México. The leaves were disinfested by immersion in sodium hypochlorite 3% for 3 min and then triple washed with sterilized water. There were five treatments: 1) antagonist 1 vs *P*. *horiana* (conidia were applied on pustules); 2) antagonist 2 vs *P*. *horiana*; 3) antagonist 1, conidia applied on healthy chrysanthemum leaves; 4) antagonist 2, conidia applied on healthy chrysanthemum leaves; and 5) control, *P*. *horiana* infected leaves treated with sterile water. Ten leaves per treatment were put into a humid chamber, each leaf representinged a repetition. A spore suspension (2×10^5^ conidia mL^-1^) of each antagonist was sprayed onto pustules on diseased leaves and onto healthy chrysanthemum leaves. All treatments were incubated at 24°C and 12 h light/dark. When signs of antagonists appeared, a sample of the fungus was cultured on synthetic PDA (Bioxon, Mexico) to confirm that it was the fungus originally inoculated.

After 96 h of incubation, antagonism percentages, measured as the proportion of pustules of *P*. *horiana* colonized by *C*. *cladosporioides* and *C*. *pseudocladosporioides*, were recorded. Differences in percentage were statistically tested by one-way ANOVA. To meet ANOVA assumptions, normal distribution was assessed by a Shapiro-Wilk test [P>0.05] and homogeneity of variance was evaluated by Levene’s test [P>0.05]. The differences among treatments were tested by post hoc Ryan-Einot-Gabriel-Welch based on an *F* test (REGW-*F*; P = 0.05). All statistical analyses were carried out using SPSS Statistics 21.0.

#### Microscope observations

Leaves collected from *Chrysanthemum* × *morifolium*, infected and non-infected with chrysanthemum white rust, were treated under the same conditions and subjected to the same treatments as in the antagonism assay described above. After 36 h of incubation, 0.5 × 0.5 mm samples of the leaves were fixed in glutaraldehyde/paraformaldehyde 3:1 (in 0.2 M phosphate buffer, pH 6.8) overnight. Fixed leaf samples were washed four times with phosphate buffer for 15 min each, dehydrated through an ethanol series (30–100%, 1 h each) and infiltrated with LR-White [40]. Sections of 1μm thickness were cut on an ultramicrotome Reichert Jung Ultra E (USA) and stained with toluidine blue 1%. For SEM, pustules were dehydrated in a CO_2_ vacuum, mounted on carbon tape and coated with gold. The samples were observed on a JEOL JSM6360LV low vacuum SEM (JEOL, USA).

#### Glucanase and chitinase production

Glucanase production of both isolates was evaluated on growth medium consisting of an agar synthetic medium (NaNO_3_, 0.2; KH_2_PO_4_, 0.1; MgSO_4_7H_2_O, 0.05; KCl, 0.05; agar 15 g/L and deionized water) supplemented with laminarin 1% (laminarin from *Laminaria digitata*, Sigma Aldrich, México) as a sole carbon source. Chitinase production was evaluated on the same synthetic basal medium but supplemented with colloidal chitin 1% rather than laminarin. Colloidal chitin was prepared using 10 g of purified crab shell chitin (Sigma Aldrich, México) suspended in 100 mL concentrated HCl for 2.5 h at 4°C and then was washed with cold deionized water and NaOH overnight at 4°C, followed by re-washing with cold deionized water at approximately pH 7.0. Mycelia discs (4 mm) were removed from PDA purified cultures with a sterilized needle and transferred to specific carbon-source media. After incubation at 24°C for 15 days, growth was evaluated either by measurement of the diameter of the developing colonies in comparison with a negative control (basal medium without carbon source), or by comparison with a positive control (basal medium supplemented with D-glucose 1%) in threefold replication.

## Results

### Morphological determination of *Cladosporium* spp.

**Fig 1:**
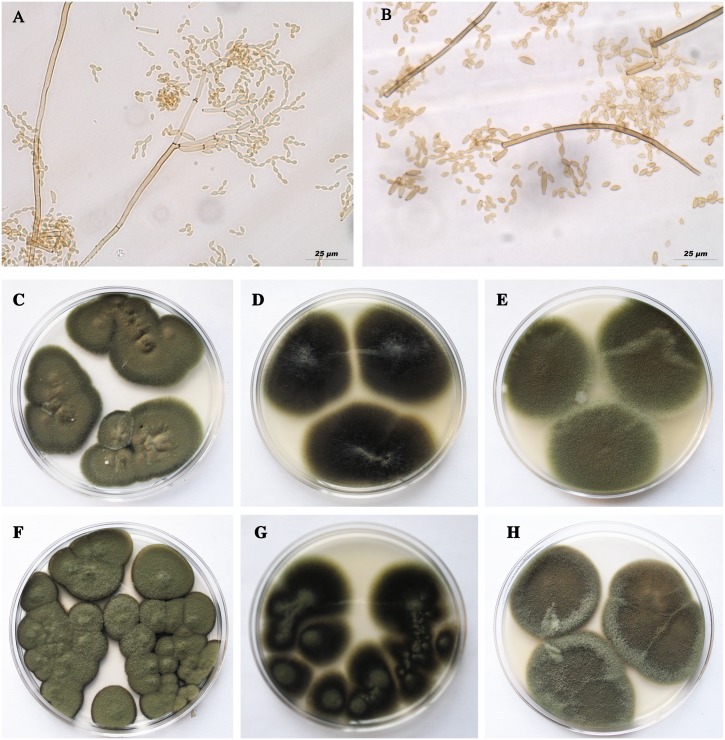
Morphological characteristics of *Cladosporium* spp. associated with *Puccinia horiana*. Light microscopy of conidiophores, ramoconidia and conidia on SNA medium. (A) *Cladosporium cladosporioides*, (B) *C*. *pseudocladosporioides*. Morphology of *C*. *cladosporioides* colonies on three different media: PDA (C), MEA (D), OA (E); *C*. *pseudocladosporioides* colonies on PDA (F), MEA (G), OA (H).

Our collections from chrysanthemum plants yielded two different strains of fungus resembling the *Cladosporium* genus (*Cladosporiaceae*, Capnodiales) [18]. Based on the morphological characteristics, one of the isolated fungi was identified as *Cladosporium cladosporioides* (Fresen.) GA de Vries, and the second isolated fungus was identified as *Cladosporium pseudocladosporioides* Bensh, Braun & Crous (Fig 1). Their morphological characteristics are summarized in Table 2.

**Table 2:** Morphological characteristics of two different *Cladosporium* species associated with *Puccinia horiana*. Conidiophore, ramoconidia and conidia characteristics were from colonies grown on SNA medium for 5 days at 24°C in the dark.

	*Cladosporium cladosporioides*	*Cladosporium pseudocladosporioides*
**REPRODUCTIVE STRUCTURES**		
**Conidiophore**	Straight, solitary, unbranched, terminal or lateral and without nodules.	Straight, solitary, unbranched, terminal or lateral and without nodules.
	3.63–**3.1**–2.77 μm	4.57–**3.44**–2.21 μm
**Ramoconidia**	Usually in groups of three or four, at the tip of conidiophores, straight, cylindrical-oblong in shape.	Usually in groups of three, at the tip of conidiophores, cylindrical-oblong in shape.
	5.47–**9.27**–18.15 × 2.43–**2.96**–3.83 μm	7.37–**13.94**–38.23 × 2.43–**3.57**–4.76 μm
**Conidia**	Numerous, in chains of up to nine conidia. They are limoniform, ovoid, obovoid to subglobose, aseptate, light brown, hila conspicuous.	Numerous, in chains of up to six conidia. They are limoniform, obovoid, ovoid to ellipsoidal in shape, aseptate, light brown, hila conspicuous.
	2.94–**4.08**–5.02 × 1.77–**2.18**–2.94 μm	4.08–**5.64**–7.59 × 1.82–**2.76**–3.97 μm
**COLONIES ON DIFFERENT GROWING MEDIA**		
**On PDA medium**	Olivaceous with aerial mycelia diffuse, floccose-felty, reverse olive-black color.	Velvety, brown-reddish with dark margins, reverse brown-reddish color.
**On MEA medium**	Olivaceous-black and floccose, reverse olivaceous-black color.	Floccose olivaceous-black, reverse olivaceous-black color.
**On OA medium**	Velvety olivaceous to grey-olivaceous color with aerial mycelia, reverse grey-olivaceous color.	Velvety grey to grey-brown, reverse greybrown color.

### Sequence analysis of *C*. *cladosporioides* and *C*. *pseudocladosporioides*, ITS, ACT & EF-1α

**Fig 2:**
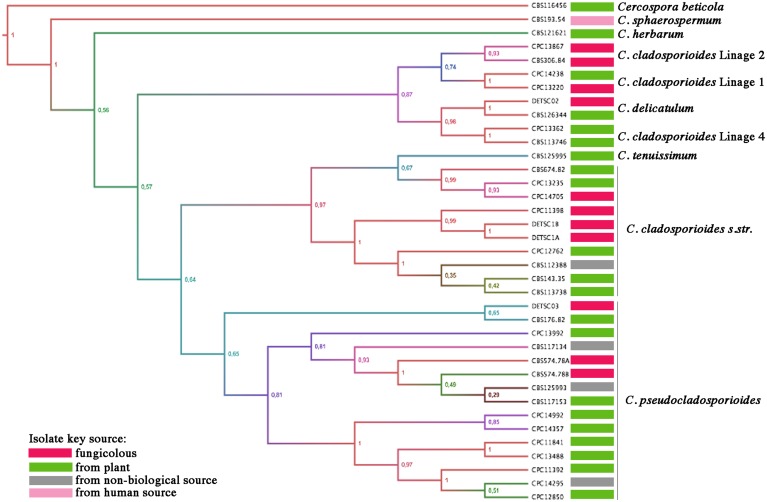
Consensus phylogram from 80 0001 trees resulting from Bayesian analysis of 43 isolates in a combined ITS, ACT & EF-1α alignment. Bayesian posterior probabilities on the tree are marked on the nodes. Isolate sources are color coded on branch tips, as indicated in the legend. The tree was rooted to sequences of *Cercospora beticola* strain CPC 11557, *Cladosporium herbarum* and *Cladosporium sphaerospermum*, representing the other two well defined phylogenetic groups within the genus. Also were included *C*. *tenuissimum* as support clade and *C*. *delicatulum* due to its presence on *P*. *horiana* pustules, but without antagonism effect.

The analysis of the genomic sequences of internal transcribed spacer (ITS) region, actin (ACT) and translation elongation factor-1α (EF-1α) supported the morphological identification of the fungal isolates as *C*. *pseudocladosporioides* and *C*. *cladosporioides*. In order to determine inter- and intra-species phylogenetic similarities, based on sequence analysis and the origin of isolates, we performed a Bayesian analysis (Fig 2). All sequences were clustered into the *Cladosporium* genus, within the *C*. *cladosporioides* complex with strong support probabilities. *Cladosporium cladosporioides*, was clustered with CPC-11398 (from *Phragmidium griseum*) in one of three well defined sub-clades at the species core (Fig 2), consistently a member of *C*. *cladosporioides*. This species is composed of one of three well defined clades clustered with *Cladosporium delicatulum*, so in consequence is polyphyletic. *Cladosporium pseudocladosporioides* was clustered with CBS-176.82 (from *Pteridium aquilinum*) in one well defined sub-clade (Fig 2); all isolates were clustered together, so corresponding to a monophyletic lineage and consequently representing a true member of *C*. *pseudocladosporioides*.

### Antagonism assay

**Fig 3:**
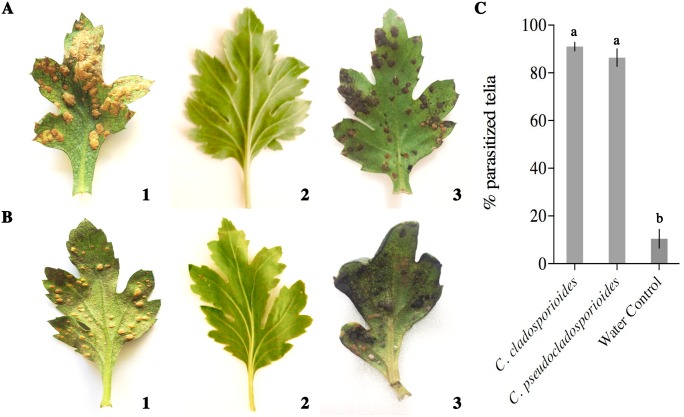
Antagonism of *Cladosporium cladosporioides* and *C*. *pseudocladosporioides* on *P*. *horiana* telia. (A) Assay with *C*. *cladosporioides*. (B) Assay with *C*. *pseudocladosporioides*. (1): Chrysanthemum leaves infected with *P*. *horiana*, without the antagonists; (2): Leaves without *P*. *horiana* and antagonist isolates applied; (3): Leaves with *P*. *horiana* and treated with the antagonists. (C) Percentage of *P*. *horiana* pustules parasitized by *Cladosporium* spp. Bars with different letters indicate significant differences at P<0.05 (REGW-F test). The line in each bar represents the standard error.

The telia of *P*. *horiana* treated only with water showed no alteration and had normal appearance (Fig 3A1 and 3B1). No *C*. *cladosporioides* or *C*. *pseudocladosporioide*s conidia or hyphae were observed on inoculated leaf surfaces in areas devoid of *P*. *horiana* pustules (Fig 3A2 and 3B2). In contrast, 96 h after inoculation with *Cladosporium* isolates, pustules showed an appearance similar to that observed on leaves from diseased plants collected in the commercial greenhouse (Figs 3A3 to 3B3 and 4). The two *Cladosporium* isolates showed significant (P<0.05) parasitism on *P*. *horiana* pustules (Fig 3C). Pure cultures obtained from the parasitized pustules inoculated with the two antagonists exhibited the same morphological characteristics as the original isolates (data not shown).

**Fig 4:**
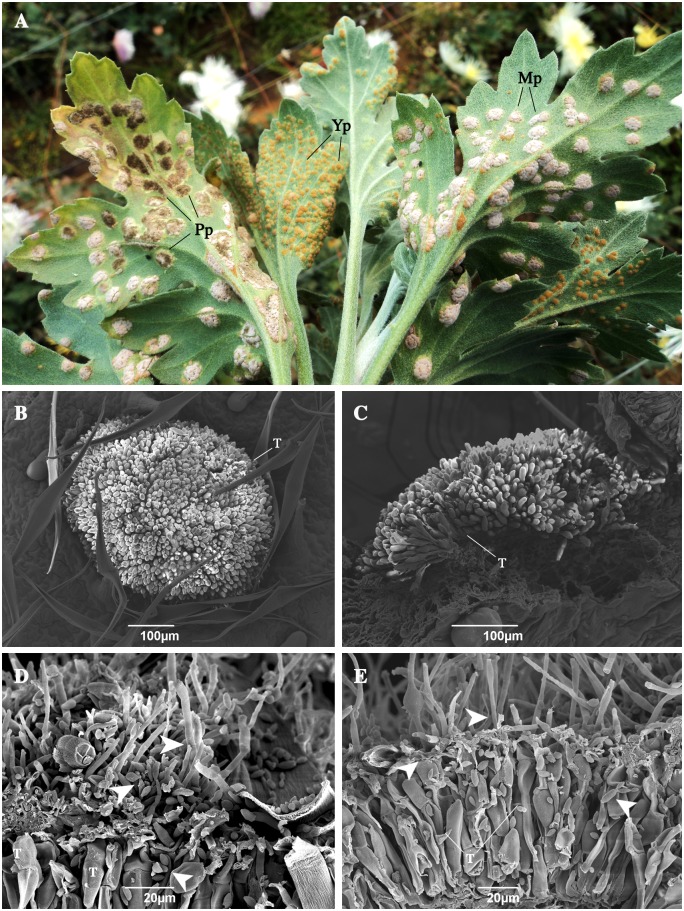
Telia of *Puccinia horiana* parasitized by fungi in the field. (A) Leaves collected from field. Pp: parasitized pustule; Yp: young pustule; Mp: mature pustule. (B-C) Undamaged pustules. (D-E) Damaged pustules with fungus morphologically resembling the *Cladosporium* genus. **Arrowheads** indicates structures resembling *Cladosporium* sp. T: teliospores of *P*. *horiana*.

### Interaction of *Cladosporium* isolates and *P*. *horiana* examined under SEM and light microscopy

**Fig 5:**
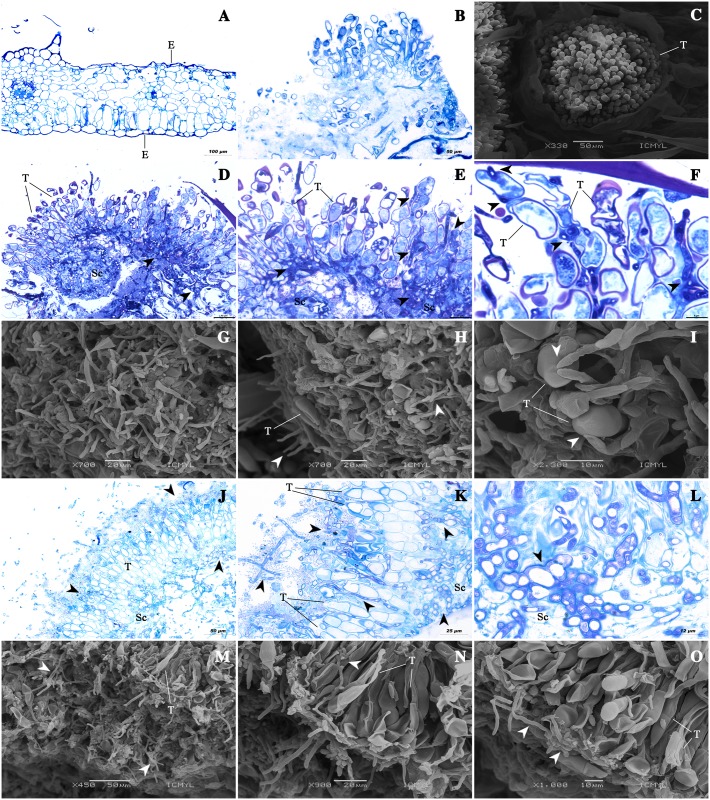
SEM and light microscopy observations of *Puccinia horiana* telia parasitized by two *Cladosporium* spp. isolates. (A) Chrysanthemum leaves without *P*. *horiana* and antagonist isolates applied; (B-C) Leaves infected with *P*. *horiana*, without the antagonists; (D-F) *C*. *cladosporioides* in interaction with *P*. *horiana* pustules, teliospores and sporogenous cells under light microscopy; (G-I) *C*. *cladosporioides* and *P*. *horiana* teliospores under SEM, showing the colonized pustule surface; (J-L) *C*. *pseudocladosporioides* in interaction with *P*. *horiana* pustules, teliospores and sporogenous cells under light microscopy; (M-O) *C*. *pseudocladosporioides* and *P*. *horiana* teliospores under SEM, showing the surface of the colonized pustule. **Arrowheads** indicates structures of the antagonist *Cladosporium* spp. **E**: leaf epidermis, and **T**: *P*. *horiana* teliospores, **Sc**: *P*. *horiana* sporogenous cells.

The morphology of *Puccinia horiana* corresponded to the recently available descriptions [6, 9, 41], and almost all plant tissue was colonized by the CWR (Fig 5B and 5C). The healthy chrysanthemum leaves were not parasitized by the *Cladosporium* spp. isolates (Figs 3 and 5A). Once *Cladosporium cladosporioides* and *C*. *pseudocladosporioide*s conidia germinated, they began to develop an intimate and active physical association with *P*. *horiana*. Teliospores from pustules colonized by *C*. *cladosporioides* (Fig 5D–5I) and *C*. *pseudocladosporioides* (Fig 5J–5O) were collapsed but no evidence of direct penetration was observed. Their cytoplasm was disrupted showing a vacuolated appearance and the wall structures were slimmed and collapsed causing the deformation of teliospores (Fig 5F and 5G for *C*. *cladosporioides* and Fig 5K, 5N and 5O for *C*. *pseudocladosporioides*). Further invasion and damage on *P*. *horiana* sporogenous cells occurred, and conidiophores of the antagonists were observed protruding from the pustules (Fig 5D–5O).

### Growth *in vitro* on glucanase and chitinase media

**Fig 6:**
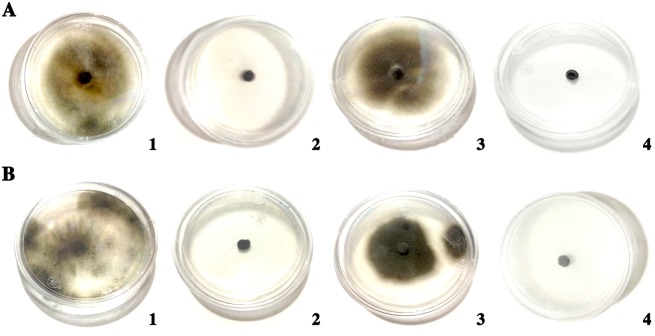
Growth of two *Cladosporium* spp. isolates on media with different carbon sources. (A) *C*. *cladosporioides*. (B) *C*. *pseudocladosporioides*. (1) laminarin 1%; (2) colloidal chitin 1%; (3) glucose 1%; (4) medium without carbon source.

On the specific nutritional carbon-source media, *Cladosporium cladosporioides* grew 49.66 ± 0.47 mm on laminarin 1%, 44.66 ± 0.47 mm on glucose 1% and has no growth on colloidal chitin 1%, in case of *Cladosporium pseudocladosporioides* grew 32.66 ± 3.29 mm on laminarin 1%, 36.00 ± 4.32 mm on glucose 1% and has no growth on colloidal chitin 1%. Both *Cladosporium* isolates grew on the medium with laminarin as a sole carbon source, but not on that prepared with colloidal chitin (Fig 6).

## Discussion

Morphological identification of *Cladosporium* spp. has been a difficult subject. Conidiophore and conidia size and shape are important characters, but usually dimensions overlap among species in the genus. However, molecular analysis has been a useful approach for the identification of *Cladosporium* species. Discrimination between *C*. *cladosporioides*, *C*. *pseudocladosporioides* and other taxa in the *C*. *cladosporioides* complex was made by Bensh *et al*., until 2010 [35], based on molecular phylogeny using ITS, ACT and EF-1α regions. These regions were used by Bensh *et al*. [18] to explain diversity and evolutionary trends in the *Cladosporium* genus. ITS alone does not give good species resolution [39] but ACT and EF-1α, in contrast, demonstrate a high degree of divergence among species [35, 42]. In the present study, we used an integrated approach based on the analysis of both molecular and morphological characters to determine the mycoparasitic species isolates as *C*. *cladosporioides* and *C*. *pseudocladosporioides*. Regarding intra-species relationships, our *C*. *cladosporioides* isolate showed some degree of diversification, as it clustered with another rust fungicolous isolate in a clearly supported clade inside core *C*. *cladosporioides*; likewise, there was strong clade support in *C*. *pseudocladosporioides* and *C*. *cladosporioides* lineages. Our results are consistent with Bensh *et al*. [35], and support the possible presence of cryptic species complexes on *C*. *pseudocladosporioides* and *C*. *cladosporioides* lineages. Both species are widely distributed and well adapted to various environments [18,35]. *Cladosporium cladosporioides* has already been reported parasitizing other fungi, such as *Venturia inaequalis* [29], *Erisyphe cichoracearum* [43], *Botrytis fabae* [44], *Sclerotinia sclerotiorum* [45] and rust fungi such as *Puccinia graminis* f.sp. *tritici* [30]. To our knowledge, *C*. *pseudocladosporioides* has not previously been reported parasitizing another fungus. This is the first report of both species potentially parasitizing *P*. *horiana* telia, and they occur naturally on this rust.

The association of both *Cladosporium* spp. on the sporogenous cells, without direct penetration of spores, was previously reported in *Cladosporium* sp. parasitizing *Exobasidium camelliae* var. *gracilis* [46], *C*. *phylophillum* on *Taphrina* sp., *C*. *exobasidii* on *Exobasidium vaccinii* and *Exobasidium warmingii*, and *C*. *epichloës* on *Epichloë typhina* [47]. This kind of relationship, without direct penetration, was probably due to differences between the teliospores and sporogenous cells of *P*. *horiana*, such as wall structural complexity between the stroma and spores, as well as different arrangement and proportion of chitin, glucans, glycoproteins, melanin and some other structural chemical compounds between different structures, as reported on *Puccinia graminis* and some other Pucciniales [48, 49, 50, 51], and it has been hypothesized that β-1,3 glucanases could be determinant for this *Cladosporium* spp. nutritional and spatial association [25]. Although there was evidence that *C*. *cladosporioides* and *C*. *pseudocladosporioides* were able to grow on laminarin media as the sole carbon source, we are not certain if these fungi excrete glucanases to parasitize *P*. *horiana*; however, the involvement of this enzyme in mycoparasitic *Cladosporium* relationships was previously reported for *C*. *tenuissimum* against *Uromyces appendiculatus* [25]. It is also possible that antibiotic mechanisms were affecting teliospore morphology and had repercussions probably in terms of potential loss of viability of teliospores, as previously reported in almost all mycoparasitic relationships of strains of *C*. *tenuissimum* [25, 26, 27], *C*. *uredinicola* [21, 22, 23], *C*. *gallicola* [28], *C*. *aecidiicola* [24] and *Cladosporium* sp. [17, 52]. Various antifungal compounds have been reported and isolated from some *C*. *cladosporioides* strains [53, 54] and *C*. *pseudocladosporioides* has shown some antibiotic activity [55].

Currently, control of chrysanthemum white rust in greenhouses and semi-covered growing systems is focused mainly on fungicides and some resistant cultivars. The potential resistance of *P*. *horiana* to fungicides represents a challenge for the development of new schemes to reduce damage by chrysanthemum white rust. Although efforts have been made to apply biological control against this disease using *Verticillium lecanii*, *Aphanocladium album* or *Cladosporium* spp. [4, 15, 16, 17], so far none of them are widely used. Since *C*. *cladosporioides* and *C*. *pseudocladosporioides* isolates altered the morphology of teliospores, and possibly reduced both viability and production, they might have potential for chrysanthemum white rust management in an integrated disease management scheme. Further studies must be carried out as, among other things, it is necessary to know the disease’s biology, ecology and mycoparasitism under controlled and commercial conditions, and to ascertain the antagonistic activity of the hyperparasites and their role in nature. In addition, it must be confirmed that these isolates do not harm other crops and that they have no adverse effects on humans or other animals. The present study provides a basis for such further studies.

## Supporting information

S1 Fig Phylograms of *Cladosporium* isolates EFα1 and ACT by Neighbour Joining method. (A) EFα1 partition; (B) ACT partition. (Using the same sequences as in Table 1).
(TIF)
