# Supplementary figures and images for: Cladosporium cladosporioides and Cladosporium pseudocladosporioides as potential new fungal antagonists of Puccinia horiana Henn., the causal agent of chrysanthemum white rust
Source: PLoS One. 2017 Jan 31;12(1):e0170782. doi: 10.1371/journal.pone.0170782 (PMC5283677; doi:10.1371/journal.pone.0170782)

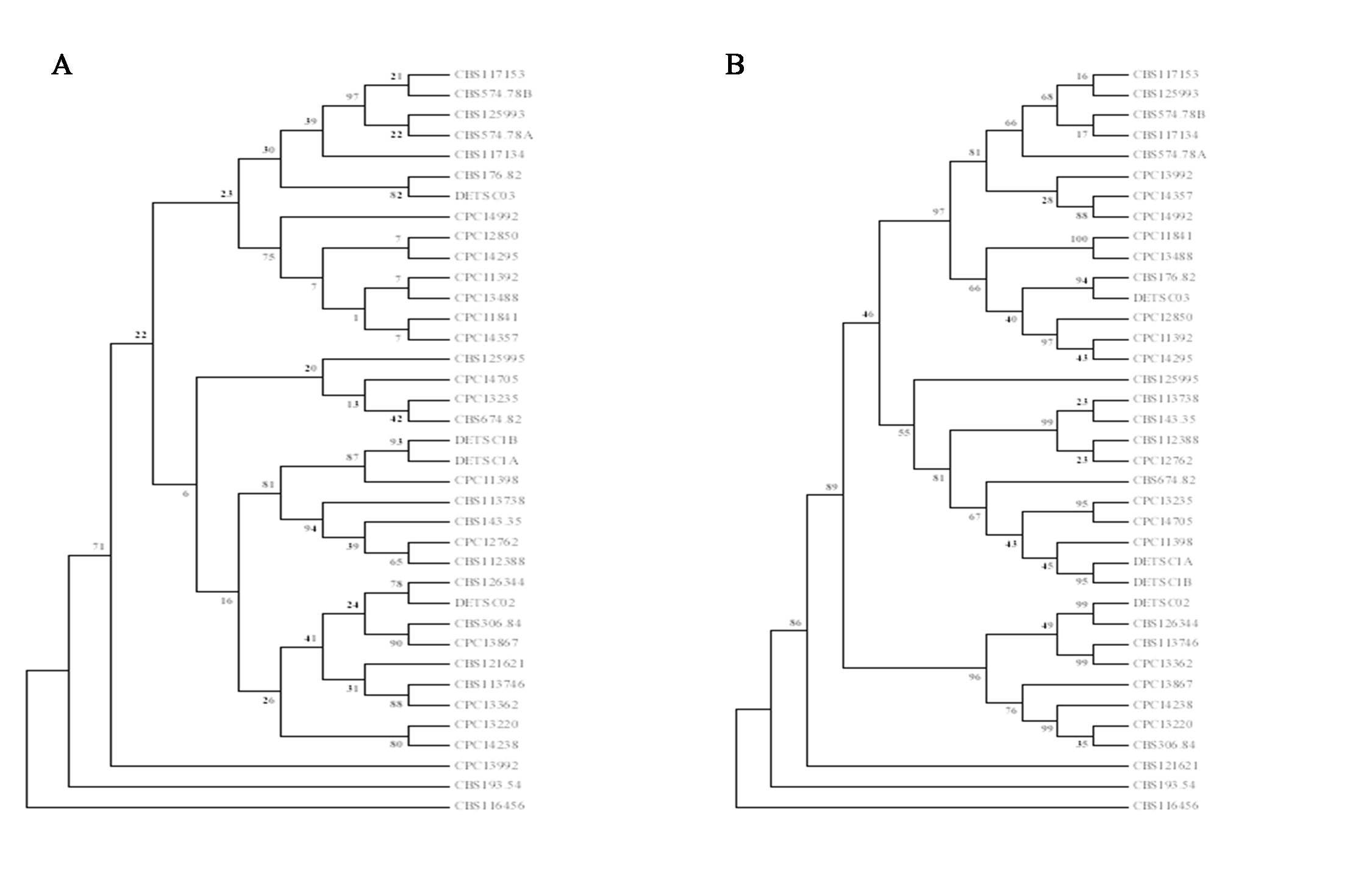

Supplement: S1 Fig — (A) EFα1 partition; (B) ACT partition. (Using the same sequences as in Table 1). (TIF) [file pone.0170782.s001.tif]
